# A Meta-Analysis of the Relationships Between Emotional Intelligence and Employee Outcomes

**DOI:** 10.3389/fpsyg.2022.611348

**Published:** 2022-04-25

**Authors:** Çaǧlar Doǧru

**Affiliations:** Department of Management and Organization, Ufuk University, Ankara, Turkey

**Keywords:** emotional intelligence (EI), organizational commitment, organizational citizen behavior (OCB), job satisfaction, job performance, job stress, meta-analysis

## Abstract

Emotional intelligence is an emerging field since the 1990s due to its important outcomes for employees. This study is a psychometric meta-analysis examining the links between emotional intelligence and organizational commitment, organizational citizenship behavior, job satisfaction, job performance, and job stress of employees. In this meta-analysis, carefully selected studies on emotional intelligence since the origin of the concept in 1990 were included along with studies examining its outcomes. For this analysis, three streams of emotional intelligence, consistent with previous meta-analyses, were considered: ability, self-report, and mixed emotional intelligence. This meta-analysis is an attempt to add to the literature by analyzing the relationships between emotional intelligence and selected employee outcomes over a period of time beginning in 1990. The three streams of emotional intelligence were separately analyzed to examine their relationship with employee outcomes. These outcomes were included in the study based on select research studies. Our study results showed that emotional intelligence and its three streams were positively related to organizational commitment, organizational citizenship behavior, job satisfaction, and job performance and negatively related to job stress.

## Introduction

Since the 1990s, the study of emotional intelligence has gained importance in disciplines such as psychology (Salovey et al., 2009), management (Prentice et al., 2020), organizational behavior (Minbashian et al., 2018), leadership (Goleman et al., 2013), education (Titrek, 2009), and marketing (Kidwell et al., 2011). This is due to the increasing value of emotional intelligence in employees. It is argued that a business that effectively manages emotions within its organization results in better performance and higher rates of return than companies that ignore emotions (Parmar, 2016). Emotions can be effectively managed in an organization by understanding employees (Pick et al., 2015), cultivating empathy (Petrovici and Dobrescu, 2014), giving them a chance to understand each other and creating a unique organizational emotional climate (Härtel et al., 2008). All these abilities, in addition to the capacity of the employees to monitor their own and others' emotions, were defined as *emotional intelligence* by Salovey and Mayer (1990). They viewed emotional intelligence as a subgroup of social intelligence, and following their continued research, they revised it and propounded the four-branch model of emotional intelligence, which included *perception and expression of emotion, assimilating emotion in thought, understanding and analyzing emotion, reflective regulation of emotion* (Mayer and Salovey, 1997). In their studies, they projected emotional intelligence as an ability, and recent research has added weight to the ability and the integrative model approaches in this field (Mayer et al., 2008).

In this study, the primary goal is to update the prior meta-analyses on the relationships between emotional intelligence in organizations and employee outcomes. Scholars have already linked particular employee outcomes with emotional intelligence. These include performance (Gong et al., 2019), job satisfaction (Feyerabend et al., 2018), organizational commitment (Baba, 2017), burnout (Hong and Lee, 2016), stress (Sarrionandia et al., 2018), leadership (Mullen et al., 2019), motivation, organizational justice, and counterproductive work behavior (Tziner et al., 2020). In this research, we have attempted to articulate the consequences of emotional intelligence in organizations by conducting a meta-analysis. Various useful meta-analyses on emotional intelligence already exist. For example, Joseph and Newman (2010) conducted an integrative meta-analysis linking emotion perception, understanding, and regulation with performance. Harms and Credé (2010) found a positive correlation between emotional intelligence and transformational and transactional leadership. O'Boyle et al. (2011) added to the literature through their three-stream approach for emotional intelligence and the relationship between the approach with job performance. Miao et al. (2017a) also used the three-stream approach to explore the connections between emotional intelligence and job satisfaction, organizational commitment, and turnover intentions. Building on previous theoretical and methodological contributions of various scholars, in this study, it was decided to explore the relationships between emotional intelligence and certain employee outcomes using a meta-analysis covering a period of 30 years. The employee outcomes that were selected for this analysis are organizational commitment, organizational citizenship behavior, job performance, job satisfaction, and job stress. These employee outcomes were selected for two reasons. First, according to the literature survey, they are the most correlated employee outcomes with emotional intelligence. Second, the three streams of emotional intelligence and the selected employee outcomes form part of future research suggestions in studies undertaken by Ashkanasy and Daus (2005), Joseph and Newman (2010), and Mattingly and Kraiger (2019).

This study also aims to add to the existing literature on emotional intelligence. First, this study includes a vast array of studies on emotional intelligence since the origin of this concept in 1990. Second, this study explores the relationship between emotional intelligence and a wide range of selected employee outcomes, namely, organizational commitment, organizational citizenship behavior, job performance, job satisfaction and job stress. These employee outcomes were carefully selected through a literature review. Third, this study adopts the three-stream classification of emotional intelligence as highlighted by Ashkanasy and Daus (2005). This study has the following structure – a detailed theoretical review followed by the hypothesis, the research methodology, the overall analysis, and finally, the results of the study. A comprehensive discussion on the results will be presented at the end of the study.

## Theoretical Background and Hypothesis Development

### Emotional Intelligence

Salovey and Mayer (1990) were the first to assess emotional intelligence (EI) as an ability of an individual to effectively manage their own and others' emotions. According to Van Rooy and Viswesvaran (2004), it included all verbal and non-verbal abilities to understand and evaluate emotions. Additionally, there are previous studies that debate whether emotional intelligence is a trait or an ability. Some scholars argue that EI is a competence (e.g., Salovey and Mayer, 1990; Austin, 2010), and some others refer to it as a trait (e.g., Bar-On, 1997; Petrides and Furnham, 2000; Petrides et al., 2007).

Based on the different approaches for emotional intelligence, different measures have been adopted to assess them. For instance, Harms and Credé (2010) and O'Boyle et al. (2011), in their studies, discussed the Bar-On Emotional Quotient Inventory (EQ-i) (1997) and the Emotional and Social Competency Inventory (Boyatzis et al., 2011) for measuring emotional intelligence as a trait. Mayer and Salovey (1997) developed and transformed EI into a four-branch model. In 2002, the Mayer-Salovey-Caruso Emotional Intelligence Test (MSCEIT) was developed (Mayer et al., 2002) and, after a year, the 141-item scale MSCEIT V2.0 was developed (Mayer et al., 2003).

Throughout this study, the three-streams approach of emotional intelligence is used. According to Ashkanasy and Daus (2005), the first stream is ability-based, the second is self-report, and the third is mixed-model. The purpose of the study is to include as many studies as possible using the three different streams and to measure emotional intelligence.

### Organizational Commitment

Organizational commitment as a concept has been very popular among organizational behavior scholars since the 1970s. It has been associated with many important employee attitudes and behaviors like employee turnover (Marsh and Mannari, 1977; Kang et al., 2015), job satisfaction (Bartol, 1979; Culibrk et al., 2018), absenteeism (Cohen and Golan, 2007), job performance (Supriyanto, 2013), role stress (Han et al., 2015), and knowledge sharing (Curado and Vieira, 2019).

Organizational commitment is indicative of the employee's recognition and acceptance of organizational circumstances (Steers, 1977). The essential characteristics of organizational commitment include approval of organizational rules, approval of objectives and values, and behaving in favor of the organization (Porter et al., 1974). Given the multidimensional structure of organizational commitment, Meyer and Allen (1991) classified the concept into affective, normative, and continuance commitment. Affective commitment is defined as the sentimental attachment employees have for their organization, and normative commitment is built on the moral obligation they feel to stay back in an organization. Continuance commitment is when the employee prefers to remain in the organization for fear of facing a negative outcome associated with leaving the organization (Allen and Meyer, 1990).

Employees with higher emotional intelligence are believed to direct their own emotions, and therefore, they might be more committed to their organizations. These kinds of employees are more resistant to emotional surges. For this reason, their intent to leave their organizations is lower when compared to employees with a lower level of emotional intelligence (Lee and Woo, 2015). Another reason is that emotionally intelligent employees are more successful in building strong social relationships in the workplace (Schutte et al., 2001). Managers, who are recognized as the agents of the organization, provide social support that increases the level of organizational commitment (Panaccio and Vandenberghe, 2009). As evidenced from the literature by Miao et al. (2017a), and Baba (2017), there is a positive correlation between EI and organizational commitment, which is our first hypothesis.

*Hypothesis 1 (H1): EI has a positive relationship with organizational commitment of employees*.

### Organizational Citizenship Behavior

Organizational citizenship behaviors (OCB) of employees are generally related to the social and psychological aspects within organizations (Organ, 1997). These behaviors mostly go beyond the formal job description in the workplace. Among these behaviors are accepting extra responsibilities and duties, working longer hours, accepting and obeying organizational rules and procedures, and helping colleagues when they need (Organ et al., 2006). These types of activities are usually not listed in the formal reward system of an organization (Organ and Lingl, 1995).

Organ (1988) classified organizational citizenship behavior into altruism, conscientiousness, sportsmanship, courtesy, and civic virtue and used each classification to define a particular behavior exerted by the employee in an organization. For example, it is *altruism* when employees tend to help colleagues when they need anything. *Conscientiousness* is related to obeying organizational rules like working hours, for instance. When employees employ constructive approaches to issues in the organization and refrain from complaining of any inconvenience, it is *sportsmanship*. It is *courtesy* when employees stop from abusing the rights of others in the organization. Lastly, *civic virtue* refers to activities that are undertaken to serve the interests of the organization, such as being a member of various committees.

Emotional intelligence is understood to reinforce the organizational citizenship behaviors of employees in an organization. This may be deducted from the results of studies that have found that employees who are good at managing their emotions are more eager to demonstrate positive behaviors in their organizations (e.g., Miao et al., 2017c; Kim and Park, 2020). Additionally, employees with high emotional intelligence tend to volunteer helping others in the workplace. Previous studies demonstrate the positive link between EI and OCB (e.g., Turnipseed and Vandewaa, 2012; Pradhan et al., 2016; Miao et al., 2018), which is the second hypothesis.

*Hypothesis 2 (H2): A positive relationship exists between EI and organizational citizenship behavior*.

### Job Satisfaction

Job satisfaction has emerged as a very popular behavioral outcome among scholars who have been trying to locate behavioral outcomes since the beginning of 1930s (e.g., Hoppock, 1935). Job satisfaction is an attitude that signals “a positive or negative evaluative judgment toward an employee's job.” (Weiss, 2002). Ever since the introduction of the concept of job satisfaction in this field, its various impacts on employees have been examined. Among them are job performance (Li et al., 2018), turnover intentions (Lu et al., 2016), job burnout (Zhang and Feng, 2011), organizational commitment (Valaei and Rezaei, 2016), and organizational citizenship behavior (Singh and Singh, 2019). According to these studies, there are positive links between job performance, organizational commitment and organizational citizenship behavior, and job satisfaction. On the contrary, job satisfaction has negative effects on turnover intentions and burnout since it is an important element that steers an individual's happiness and enthusiasm to perform in the workplace (Piccolo et al., 2005).

Emotional intelligence is a vital input for employees feeling job satisfaction. For example, Anari (2012), in his study on high-school teachers, established positive links between emotional intelligence and job satisfaction. Similarly, Brunetto et al. (2012) found that EI was the main indicator for predicting job satisfaction in a study among 193 police officers in Australia. Furthermore, in their meta-analysis, Miao et al. (2017b) revealed that job satisfaction was positively affected by emotional intelligence regardless of gender, age, or tenure, which is the basis of our third hypothesis.

*Hypothesis 3 (H3): EI has a positive link with job satisfaction*.

### Job Performance

Job performance, in general, can be defined as the employee's activities and behaviors that directly or indirectly contribute to the organizational goals (Borman and Motowidlo, 1993). From this perspective, the level of job performance is a valuable indicator for many human resource management decisions (e.g., training and development, compensation, and promotion).

Most studies categorize job performance as a task or a contextual performance (e.g., Borman and Motowidlo, 1997; Van Scotter, 2000). Task performance includes the degree to which employees meet the standards of core and technical tasks and duties. Alternatively, contextual performance measures the degree of employees' behaviors that promote the social and psychological environment in the organization, such as helping others, taking extra responsibilities in the workplace, and obeying organizational rules and procedures (Motowidlo and Van Scotter, 1994). There are many studies that substantively establish that emotional intelligence is a meaningful precursor for performance. For example, Farh et al. (2012) found in their study on 212 professionals from different organizations that overall emotional intelligence led to more effective teamwork and higher job performance. Similarly, Li et al. (2018) found a positive correlation between trait emotional intelligence and performance among 881 teachers and 37 principals from primary schools in China. Also, O'Boyle et al. (2011) found positive correlations between all the three streams of emotional intelligence and job performance in their meta-analysis, which is our fourth hypothesis.

*Hypothesis 4 (H4): EI is positively related to job performance*.

### Job Stress

Job stress is a deviation from the ordinary psychological state of an employee due to job-related factors (Schuler, 1980). Job stress is mostly associated with poor job performance (Siu, 2003), low motivation (Luo, 1999), low job satisfaction (Parker and DeCotiis, 1983), high emotional exhaustion (Griffin et al., 2010), and high turnover intentions (Mullen et al., 2018). In general, building strong social relationships, having role clarity, providing organizational support, and encouraging knowledge sharing help employees decrease their stress levels.

In addition to environmental and organizational factors, the employees' personality, perceptions, and emotions are significant factors contributing to job stress among them (Spector and Goh, 2001; Sur and Ng, 2014). It is evident that employees who are good at managing their emotions experience lower job stress (Mann, 2004). However, it is important to note the link between emotional intelligence and job stress. Lee (2010) found a negative relationship between emotional intelligence and job stress among 152 nurses from 4 hospitals in Korea. Similarly, Shukla and Srivastava (2016) found a negative relationship between trait emotional intelligence and job stress among 564 retail employees, which is our fifth hypothesis.

*Hypothesis 5 (H5): EI is negatively related to job stress*.

## Meta-Analytical Research Methodology

### Literature Review

Since the aim of this study was to include all the relevant research so far, 1990 was chosen as the beginning year, given that it was in 1990 that Salovey and Mayer conceptualized EI. The time period for this analysis was from 1990 to 2019. However, to expand the scope of this study, studies that were published in the early months of the 2020 were also included. To increase the likelihood of identifying relevant studies, both published and unpublished research works in English were included in the analysis. Keywords such as emotional intelligence, emotional ability, emotional competency, emotional stability, organizational commitment, organizational citizenship behavior, job satisfaction, job performance, job stress, and occupational stress were used in this analysis.

To expand the scope of this study, several research techniques were adopted which were similar to those adopted in previous meta-analytic studies that were part of the literature review. First, the main electronic databases such as ABI/INFORM Global, APA PsycInfo, EBSCOhost, Google Scholar, JSTOR, ProQuest, ProQuest Dissertation and Theses, ScienceDirect, and Web of Science were scanned. Second, a further scanning was carried out by searching the archives of leading journals such as the Academy of Management Annals, the Academy of Management Journal, the Academy of Management Review, Administrative Science Quarterly, the Journal of Applied Psychology, the Journal of Management, the Journal of Organizational Behavior, the Journal of Occupational and Organizational Psychology, Leadership Quarterly, Personnel Psychology, and Personality and Individual Differences. Third, proceedings of leading conferences on Management and Psychology were also scanned (e.g., Annual Meeting of Academy of Management, European Academy of Management Conference, and the Society for Industrial and Organizational Psychology Annual Conference). This broad scanning resulted in identifying 287 articles and 118 unpublished dissertations and conference papers for examining the links between EI and organizational commitment, organizational citizenship behavior, job satisfaction, job performance and job stress. For the articles to be useful for this analysis, some inclusion criteria were determined.

### Inclusion Criteria

In order to be included in this meta-analysis, the identified studies needed to meet some rules and standards. The first criterion for any study to be included in this analysis was that it should be a quantitative empirical study providing at least correlation coefficients in its variables. The second criterion was that it should have been published between 1990 and 2020 (the first 2 months). The third criterion was that English should be the article's language. The fourth criterion was related to the sample – only studies that used unique samples when studying more than one sample were included in the analysis. This inclusion criterion was developed to prevent duplication in samples. Drawing on the recommendations from Ashkanasy and Daus (2005) and meta-analysis by O'Boyle et al. (2011), emotional intelligence was coded based on three streams (ability EI, self-report EI, and mixed EI). After screening the identified articles using the inclusion criteria, the final total sample for this meta-analysis consisted of 253 effect sizes representing data from 78,159 participants.

### Visualization of the Inclusion and Exclusion Process

**Figure 1:**
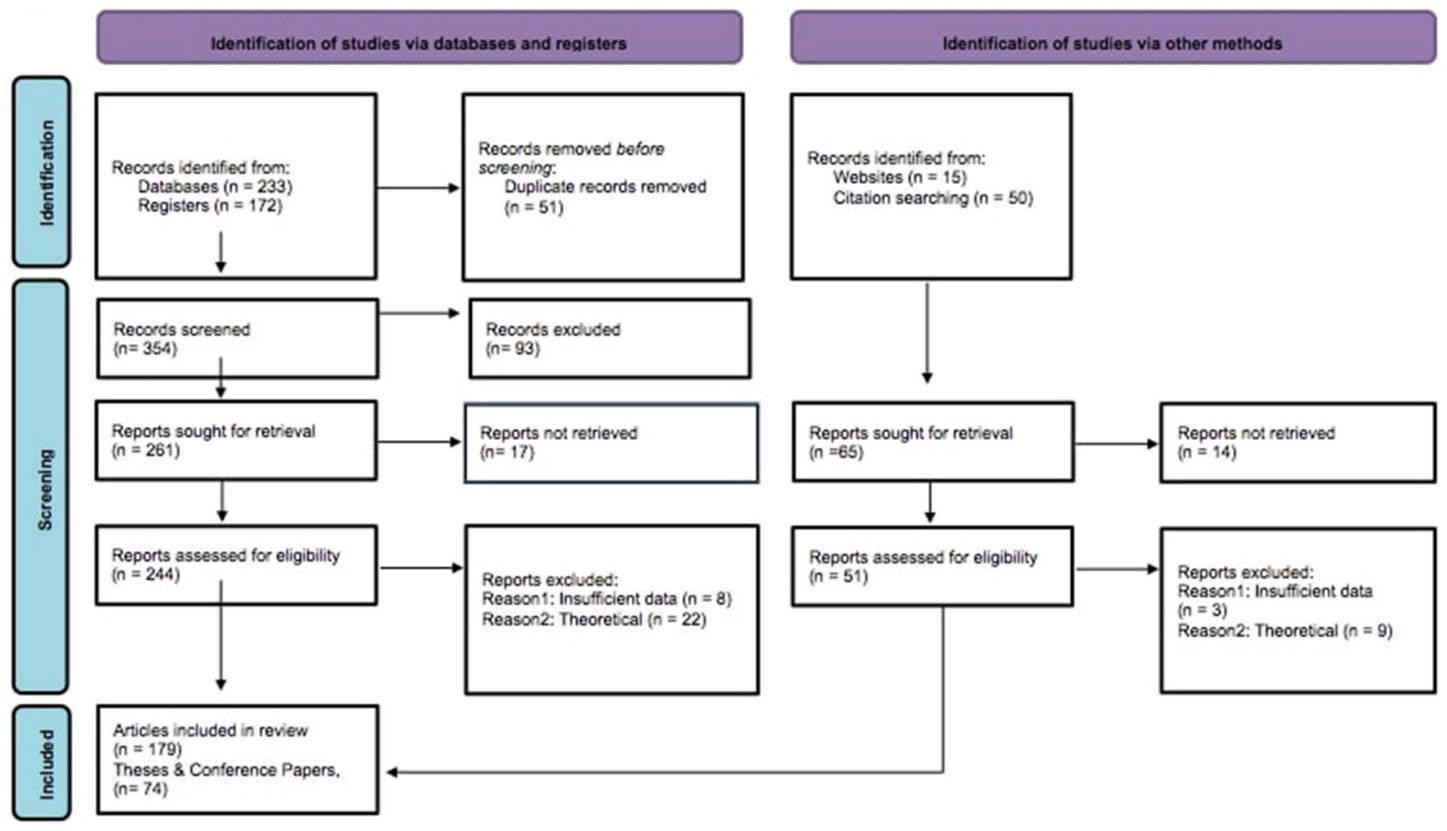
PRISMA flow diagram for new systematic reviews. Source: Page et al. (2021).

After carefully screening the existing literature on emotional intelligence and its possible outcomes in the workplace and checking the identified studies against the inclusion criteria, some studies were excluded from the analysis. In order to demonstrate the screening and the selection processes, a widely used visualization technique in meta-analyses, PRISMA Flow Diagram for new Systematic Reviews (Page et al., 2021), was employed throughout this meta-analysis and it is shown in Figure 1.

### Descriptive Statistics for the Sample

To understand the profile of the participants in the studies and to provide more information about the sample, some of the descriptive statistics were categorized on the basis of participants' gender, age, and job positions (managerial or non-managerial) as well as the publication details (year and country) of the studies. The descriptive statistics are presented in Table 1.

**Table 1:** Descriptive statistics of the samples included in the analysis.

	***f***	***%***
**Participants**		
Gender		
Female	37,516	48
Male	32,045	41
Missing data^*^	8,598	11
Total	78,159	100
Job position		
Managerial	18,759	24
Non-Managerial^**^	59,400	76
Total	78,159	100
Age		
18–30	14,850	19
31–40	24,229	31
41–50	17,195	22
50 +	11,724	15
Missing data^*^	10,161	13
Total	78,159	100

* *There is no gender or age information of the participants*.

** *When there is no information about the job position of participant employees, they are assumed to have non-managerial positions*.

### Procedure

For this study, the psychometric meta-analysis method was used. The strength of this method is that it provides a basis for estimating the variance of sampling error and gives an opportunity to estimate reliability for studies in which no reliability had been reported (Hunter and Schmidt, 2004). This method has been used in previous meta-analyses (e.g., Harms and Credé, 2010; O'Boyle et al., 2011). One of the reasons for choosing this technique is that it helps to forecast the variance associated with sampling error and artifacts. To generate artifact distributions, reliability estimates were employed to fill the gaps stemming from the absence of reliability data in some of the studies. Hunter and Schmidt (1990) suggested that the distributions of correlations were corrected in this study. Further, *r*obs and *SD*obs were corrected to help understand the artefactual biases and moderators, as done previously by Harms and Credé (2010). Using the technique proposed by Hunter and Schmidt (1990) and successfully applied by their successors (Ones et al., 1993), several sets of artifact distributions along with their descriptive details are presented in Table 2. Next, to indicate the significance of effect sizes, the confidence interval was chosen as 95% (corrected). Finally, within this scope, the sample sizes and uncorrected coefficients were converted into corrected correlation coefficients.

**Table 2:** Descriptive statistics of artifact distributions for correcting validities.

**Organizational commitment**	**Mean**	***SD***	**Mean square roots**	**SD square roots**
Predictor reliabilities	0.83	0.09	0.91	0.05
Criterion reliabilities	0.87	0.13	0.93	0.07
Range restriction values^a^	0.80	0.15	–	–

*SD, standard deviation*.

a *The ratio of the standard deviation of the selected group to the standard deviation of the referent group*.

As seen in Table 2, the overall mean of the predictor reliability for artifact distribution is 0.83 and the standard deviation value is 0.09. The mean of the square roots of predictor reliabilities is 0.91 and the standard deviation of the square roots is 0.05. The overall mean of the criterion reliabilities is 0.87 with a standard deviation value of 0.13. The mean of the square roots of criterion reliabilities is 0.93 and the standard deviation of the square roots of reliability is 0.07. Finally, the mean value of the range restriction value is 0.80 with a standard deviation value of 0.15.

## Results

After conducting the psychometric meta-analysis (Hunter and Schmidt, 2004), the results obtained from the analysis were listed separately. Beginning with the relationship between EI and organizational commitment, the results are presented in Table 3.

**Table 3:** Meta-analytic results of the relationship between EI and organizational commitment.

**Organizational commitment**	***k***	***n***	** $\bar{r}$ **	**ρ**	***SD_**ρ**_***	**95% CI lower limit**	**95% CI upper limit**
Ability EI	8	958	0.19	0.22	0.13	0.11	0.33
Self-report EI	17	3,985	0.25	0.28	0.15	0.13	0.43
Mixed EI	12	2,922	0.24	0.27	0.12	0.19	0.35
Overall EI	37	7,865	0.23	0.26	0.14	0.12	0.42
Managerial employees	11	2,136	0.30	0.32	0.09	0.21	0.43
Non-Managerial employees	21	4,271	0.22	0.24	0.07	0.18	0.29
Published studies	29	6,144	0.28	0.31	0.11	0.25	0.37
Unpublished studies	8	1,721	0.18	0.21	0.09	0.16	0.26

*EI, emotional intelligence; k, number of independent samples; n, sample size; $\bar{r}$, uncorrected sample size weighted mean correlation; ρ, corrected correlation; SD_ρ_, standard deviation of corrected correlation; CI, confidence interval*.

As evident from Table 3, according to 37 independent overall EI samples, EI is positively and significantly correlated with organizational commitment (ρ = 0.26, *p* < 0.001). Therefore, according to the result, H_1_ is supported. Additionally, all three streams of EI are also positively correlated with organizational commitment. Although there is a slight difference in magnitude, the most powerful positive relationship exists between self-report emotional intelligence and organizational commitment (ρ = 0.28, *p* < 0.001). The weakest relationship is between ability emotional intelligence and organizational commitment (ρ = 0.22, *p* < 0.001). The results of the relation between EI and organizational citizenship behavior are presented in Table 4.

**Table 4:** Meta-analytic results of the relationship between EI and OCB.

**Organizational citizenship behavior**	***k***	***n***	** $\bar{r}$ **	**ρ**	***SD_**ρ**_***	**95% CI lower limit**	**95% CI upper limit**
Ability EI	11	3,520	0.27	0.29	0.17	0.20	0.38
Self-report EI	19	5,186	0.34	0.37	0.21	0.16	0.58
Mixed EI	13	4,073	0.32	0.35	0.13	0.23	0.47
Overall EI	43	12,779	0.33	0.36	0.20	0.18	0.54
Managerial employees	15	2,957	0.25	0.27	0.14	0.23	0.31
Non-Managerial employees	23	6,109	0.36	0.38	0.16	0.22	0.54
Published studies	32	8,963	0.29	0.32	0.10	0.18	0.46
Unpublished studies	11	3,816	0.38	0.40	0.19	0.33	0.47

*EI, emotional intelligence; OCB, organizational citizenship behavior; k, number of independent samples; n, sample size; $\bar{r}$, uncorrected sample size weighted mean correlation; ρ, corrected correlation; SD_ρ_, standard deviation of corrected correlation; CI, confidence interval*.

Based on the results obtained from 43 samples, it is evident that emotional intelligence has a positive relationship with organizational citizenship behavior (ρ = *0.36, p* < 0.001). For this reason, H_2_ is supported. As with organizational commitment, self-report emotional intelligence has a strong positive relationship with organizational citizenship behavior (ρ = 0.37, *p* < 0.001). Also, as found in previous studies, the important relationship between emotional intelligence and job satisfaction was reaffirmed. Table 5 provides the correlations and additional statistical results.

**Table 5:** Meta-analytic results of the relationship between EI and job satisfaction.

**Job satisfaction**	***k***	***n***	** $\bar{r}$ **	**ρ**	***SD_**ρ**_***	**95% CI lower limit**	**95% CI upper limit**
Ability EI	16	4,761	0.21	0.24	0.32	0.10	0.38
Self-report EI	33	8,278	0.27	0.31	0.11	0.19	0.43
Mixed EI	25	6,830	0.25	0.30	0.17	0.20	0.39
Overall EI	74	19,869	0.25	0.29	0.24	0.15	0.43
Managerial employees	17	4,368	0.19	0.21	0.15	0.16	0.26
Non-Managerial employees	42	9,281	0.31	0.33	0.19	0.22	0.44
Published studies	64	16,592	0.31	0.35	0.13	0.24	0.46
Unpublished studies	10	3,277	0.20	0.24	0.21	0.18	0.31

*EI, emotional intelligence; k, number of independent samples; n, sample size; $\bar{r}$, uncorrected sample size weighted mean correlation; ρ, corrected correlation; SD_ρ_, standard deviation of corrected correlation; CI, confidence interval*.

According to 74 independent samples, emotional intelligence is positively related to job satisfaction (ρ = 0.29, *p* < 0.001). This indicates that H_3_ is also supported. The three streams of EI are also positively correlated with job satisfaction. This reaffirms another important relationship between emotional intelligence and job performance that this analysis sought to verify. The results are presented in Table 6.

**Table 6:** Meta-analytic results of the relationship between EI and job performance.

**Job performance**	***k***	***n***	** $\bar{r}$ **	**ρ**	***SD_**ρ**_***	**95% CI lower limit**	**95% CI upper limit**
Ability EI	14	5,100	0.24	0.28	0.22	0.18	0.38
Self-report EI	31	10,438	0.28	0.33	0.31	0.09	0.55
Mixed EI	23	7,731	0.27	0.31	0.19	0.13	0.49
Overall EI	68	23,269	0.26	0.30	0.28	0.10	0.49
Managerial employees	21	3,298	0.32	0.33	0.17	0.25	0.41
Non-Managerial employees	32	11,782	0.38	0.40	0.20	0.29	0.51
Published studies	59	19,127	0.21	0.25	0.15	0.17	0.33
Unpublished studies	9	4,142	0.30	0.34	0.21	0.22	0.46

*EI, emotional intelligence; k, number of independent samples; n, sample size; $\bar{r}$, uncorrected sample size weighted mean correlation; ρ, corrected correlation; SD_ρ_, standard deviation of corrected correlation; CI, confidence interval*.

As seen in Table 6, for measuring overall EI, 68 samples were used. Again, both overall emotional intelligence (ρ = 0.29, *p* < 0.001) and the three streams of EI were positively related to job performance. Therefore, H_4_ is also supported. Finally, the relationship between emotional intelligence and job stress is presented in Table 7.

**Table 7:** Meta-analytic results of the relationship between EI and job stress.

**Job stress**	***k***	***n***	** $\bar{r}$ **	**ρ**	***SD_**ρ**_***	**95% CI lower limit**	**95% CI upper limit**
Ability EI	8	2,196	−0.37	−0.42	0.16	−0.55	−0.29
Self-report EI	13	6,964	−0.41	−0.45	0.27	−0.64	−0.26
Mixed EI	10	5,217	−0.33	−0.37	0.20	−0.45	−0.29
Overall EI	31	14,377	−0.39	−0.43	0.22	−0.49	−0.37
Managerial employees	10	2,546	−0.27	−0.30	0.25	−0.32	−0.28
Non-Managerial employees	16	7,630	−0.45	−0.47	0.18	−0.68	−0.26
Published studies	25	11,230	−0.44	−0.48	0.10	−0.63	−0.33
Unpublished studies	6	3,147	−0.34	−0.38	0.21	−0.49	−0.27

*EI, emotional intelligence; k, number of independent samples; n, sample size; $\bar{r}$, uncorrected sample size weighted mean correlation; ρ, corrected correlation; SD_ρ_, standard deviation of corrected correlation; CI, confidence interval*.

It is evident in Table 7 that based on the results obtained from 31 samples, a negative relationship exists between emotional intelligence and job stress (ρ = −0.43, *p* < 0.001). This significant and negative relationship is marginally stronger than the other relationships in this study. Therefore, H_5_ is also supported. Yet again, all three types of EI were significantly related to job stress. It can be inferred that emotional intelligence is an important source for overcoming job stress in the workplace.

### Effects of Possible Moderators

The results obtained from the analysis of this meta-analysis suggested conducting a moderator analysis. To understand the effects of the substantive moderators, the moderating effects of different emotional intelligence types, managerial and non-managerial positions and publication types were analyzed by conducting separate meta-analyses.

#### Effects of Types of Emotional Intelligence

As previously stated, the potential moderating effects of ability emotional intelligence, self-report emotional intelligence, and mixed emotional intelligence were further studied by conducting separate meta-analyses. The separate results are indicated in Tables 3–7. According to the results, *Ability EI, Self-report EI*, and *Mixed EI* have similar positive and statistically meaningful effects on organizational commitment, organizational citizenship behavior, job satisfaction, and job performance but have negative effects on job stress (i.e., ρ_AbilityEI_ = 0.22; ρ_Self−report EI_ = 0.28; ρ_Mixed EI_ = 0.27 for organizational commitment; ρ_AbilityEI_ = 0.29; ρ_Self−report EI_ = 0.37; ρ_Mixed EI_ = 0.35 for organizational citizenship behavior; ρ_AbilityEI_ = 0.24; ρ_Self−report EI_ = 0.31; ρ_Mixed EI_ = 0.30 for job satisfaction; ρ_AbilityEI_ = 0.28; ρ_Self−report EI_ = 0.33; ρ_Mixed EI_ = 0.31 for job performance; and ρ_AbilityEI_ = −0.42; ρ_Self−report EI_ = −0.45; ρ_Mixed EI_ = −0.37 for job stress).

#### Effects of Managerial/Non-Managerial Positions

Few of the studies included in this meta-analysis had further categorized the employees as holding either managerial or non-managerial positions in their organizations. Employees such as branch managers, coaches, supervisors, and chief officers were categorized under managerial staff, while frontline employees and subordinates were categorized under non-managerial staff. To examine the moderating effects of managerial and non-managerial positions on employee outcomes, separate meta-analyses were conducted. According to the results of the meta-analyses, a higher correlation exists between emotional intelligence and organizational commitment when employees held managerial positions (ρ_managerial_: 0.32 > ρ_*non*−managerial_: 0.24), as shown in Table 3. On the other hand, as indicated in Table 4, the correlation between emotional intelligence and organizational citizenship behavior was lower among managers (ρ_managerial_: 0.27 < ρ_*non*−managerial_: 0.38). Table 5 shows the lower levels of correlation between emotional intelligence and job satisfaction among managers (ρ_managerial_: 0.21 < ρ_*non*−managerial_: 0.33). The same is applicable for the managers' relationship between emotional intelligence and job performance, as evident in Table 6 (ρ_managerial_: 0.33 < ρ_*non*−managerial_: 0.40). Finally, in Table 7, the negative correlation between emotional intelligence and job stress is established; however, it is stronger among employees in non-managerial positions (ρ_managerial_: −0.30 < ρ_*non*−managerial_: −0.47).

#### Effects of Publication Type

To examine the moderating effects of publication types included in this meta-analysis, both published and unpublished studies were included in separate analyses. This was done to overcome the “file drawer problem” (Harms and Credé, 2010), given that most of the results in this analysis were derived from published studies. According to the results, the correlations between the variables differ based on whether a study is published or unpublished. For example, in Tables 3–7, the corrected correlation between emotional intelligence and organizational commitment in published studies is higher than the one in unpublished studies (ρ_published_: 0.31 > ρ_*unpublished*_: 0.21). Similarly, between emotional intelligence and job satisfaction (ρ_published_: 0.35 > ρ_*unpublished*_: 0.24) and between emotional intelligence and job stress (ρ_published_: −0.48 > ρ_*unpublished*_: −0.38), the same correlation exists. However, the corrected correlation between emotional intelligence and organizational citizenship behavior in published studies is lower than the one in unpublished studies (ρ_published_: 0.32 < ρ_*unpublished*_: 0.40). Similarly, the correlation between emotional intelligence and job performance is lower in published studies (ρ_published_: 0.25 < ρ_*unpublished*_: 0.34).

## Discussion

### Findings and Theoretical Contributions

With the help of this analysis, the relationships between EI and selected employee outcomes in organizations are presented herewith. According to the results obtained in this study, emotional intelligence and its three streams are positively related to organizational commitment, organizational citizenship behavior, job satisfaction, and job performance; however, they are negatively related to job stress. If the relationship between the different streams of EI and organizational commitment is analyzed, it is noticed that self-report EI is slightly stronger than mixed EI and ability EI (ρ_Self−report EI:_ 0.28 > ρ_*Mixed EI*:_ 0.27 >ρ_*Ability EI*:_ 0.22). Similarly, the relationship between the different streams of EI and organizational citizenship behavior shows that self-report EI is slightly stronger than ability EI and mixed EI (ρ_Self−report EI:_ 0.37 > ρ_*Mixed EI*:_ 0.35 >ρ_*Ability EI*:_ 0.29). Additionally, self-report EI is slightly stronger than ability EI and mixed EI when there exists a relationship between the different streams of EI and job satisfaction (ρ_Self−report EI:_ 0.31 > ρ_*Mixed EI*:_ 0.30 >ρ_*Ability EI*:_ 0.24). In the relationship between EI streams and job performance, self-report EI is still stronger than mixed EI, and ability EI is the weakest (ρ_Self−report EI:_ 0.33 > ρ_*Mixed EI*:_ 0.31 >ρ_*Ability EI*:_ 0.28). However, when samples of job stress is analyzed, although self-report EI has the strongest negative correlation, ability EI emerges second (ρ_Self−report EI:_ −0.45 > ρ_*Ability EI*:_ −0.42 > ρ_*Mixed EI*:_ −0.37). These results can be used to explain the ranking within the three streams of emotional intelligence. In general, except for the relationships between the EI streams and job stress, it is evident that self-report EI is the most influential among all three EI streams. Although it is useful to note that the differences in their magnitudes are quite slim, in the relationships between the EI streams and job stress alone, ability EI ranked second while mixed EI ranked third.

When the results of this meta-analysis are compared with the previous meta-analyses, it is evident that the findings of the relationships between EI and organizational commitment are consistent with surveyed literature. Miao et al. (2017a) also found a positive correlation between self-report EI and organizational commitment, which is slightly stronger (ρ = 0.43) than the result obtained in this study (ρ = 0.28). Their result on mixed emotional intelligence is also higher (ρ = 0.43) than the one in this study (ρ = 0.27). Previous meta-analyses also found a positive correlation between EI and organizational citizenship behavior. For example, Miao et al. (2017c) obtained positive correlations between the three streams and organizational citizenship behavior. The corrected correlation coefficients in this analysis are marginally lower than their results. There are also similarities between this research and the analysis of Miao et al. (2017a) on the link between emotional intelligence and job satisfaction. Furthermore, the results obtained from this meta-analysis indicate a positive link between EI and job performance; these results are consistent with the previous meta-analysis of O'Boyle et al. (2011). The last relationship examined in this meta-analysis was between EI and job stress. The negative relationship between them was already identified in the studies that were included in this study. Since there was no meta-analysis in the literature that examined this relationship, the results of this study were consistent with the results of separate studies (e.g., Mikolajczak et al., 2007; Karimi et al., 2014).

Finally, it is important to flag the effects of managerial and non-managerial positions of the employees on the relationships between emotional intelligence and employee outcomes. As reported in the results shared above, it is evident that, when employees hold managerial positions, their emotional intelligence positively influences their level of organizational commitment and a stronger correlation is obtained (ρ_managerial_: 0.32 > ρ_*non*−managerial_: 0.24). However, correlations between emotional intelligence and organizational citizenship behavior (ρ_managerial_: 0.27 < ρ_*non*−managerial_: 0.38), job satisfaction (ρ_managerial_: 0.21 < ρ_*non*−managerial_: 0.33), job performance (ρ_managerial_: 0.33 < ρ_*non*−managerial_: 0.40), and job stress (ρ_managerial_: −0.30 < ρ_*non*−managerial_: −0.47) was weaker in those employees holding managerial positions. From this perspective, although there are positive relationships between emotional intelligence and organizational citizenship behavior, job satisfaction, and job performance, it is evident that those employees who hold non-managerial positions exhibit stronger positive correlations to these outcomes. A similar trend is observed in the negative relationship between emotional intelligence and job stress among non-managers in the workplace.

In this study, an attempt was made to add to the existing literature on emotional intelligence by determining the nature of relationships between emotional intelligence and selected employee outcomes such as organizational commitment, organizational citizenship behavior, job satisfaction, job performance, and job stress. These relationships were distinguished by ability EI, self-report EI, and mixed EI. This helped us to see the consequences of emotional intelligence on employees in a more detailed way. Lastly, the categorization of managerial and non-managerial roles in the samples provided valuable insights into the relationships between emotional intelligence and employee outcomes.

### Limitations and Future Research Suggestions

One of the limitations was the methodology used in the studies. Some studies used self-reports for organizational citizenship behavior and job performance. Though these studies were few, their inclusion in this analysis is a limitation for more accurate results. Another limitation is the inclusion of unpublished studies such as dissertations in the analysis. Yet again, though there were few dissertations compared to other published resources, it is important enough to be flagged as a limitation for this analysis. The third limitation was that only English sources were included in the analysis. Finally, moderators and contextual factors were not included to retain the focus on the aim of the research.

The limitations listed in this meta-analysis provide a basis for future research in this area. Researchers should also consider including more moderators and contextual factors while assessing the outcomes of emotional intelligence in their future studies. Future research should also examine the effects of emotional intelligence on other factors like leadership, occupational stress, role stress, innovative behavior and social relations. Another potential variable that has been largely underemphasized is the correlation between emotional intelligence and digital transformation in the workplace (e.g., Kaur and Sharma, 2021). Thus, researchers should investigate the role of emotional intelligence on the future of work and employees' perceptions of digitalization in the workplace (e.g., Stubbemann, 2021).

### Practical Implications

Emotional intelligence gains importance day by day for human resource managers and line managers. In general, human resource managers are more eager to select and place candidates with higher emotional intelligence (Chia, 2005). Similarly, line managers are satisfied with the performance of employees with higher emotional intelligence (Gong et al., 2019). This is because these employees can manage their own emotions as well as their colleagues' emotions. With the help of emotional intelligence, employees' satisfaction from job (Soleimani and Einolahzadeh, 2017), organizational commitment (Jain and Duggal, 2018), and job performance (Joseph et al., 2015) is set to increase. For these reasons, human resource departments should plan strategies for increasing the emotional intelligence of their employees. They could design training and development programs to increase ability EI, self-report EI, and mixed EI. Human resources managers could also set rules and standards for rewarding employees with favorable behaviors in the workplace. In addition, line managers could demonstrate effective leadership by promoting employee outcomes based on emotional intelligence.

## Conclusion

This meta-analysis was an attempt to explore the consequences of emotional intelligence on employee outcomes with the help of previous studies within a time frame of the last 30 years. From this perspective, this study has tried to add an important brick on the wall of emotional intelligence literature. Consistent with the previous meta-analyses, the three-stream approach for emotional intelligence was adopted for this study as well. After carefully examining the studies, it has been observed that all streams of EI are positively related to organizational commitment, organizational citizenship behavior, and job satisfaction whereas they are negatively related to job stress. According to the results of this meta-analysis, the magnitudes of the correlations were higher in self-report emotional intelligence compared to ability emotional intelligence; however, the differences were not very large.

From this comprehensive meta-analysis, it can be inferred that employees who are good at managing their own emotions and their colleagues' emotions are more committed to their organizations and are more eager to show organizational citizenship behavior, evince job satisfaction, and evince better job performance, and their level of job stress tends to decrease. Since these are all favorable employee outcomes, managers should design development programs for increasing the capacity of emotional intelligence among employees in the organization. In addition to other job-specific competencies, they should select and place employees with high emotional intelligence.

By including all three streams of emotional intelligence to examine their links with employee outcomes, this holistic meta-analysis is a first step for future studies exploring important relationships and developing research models on emotional intelligence. Although there are comprehensive studies in the literature, more studies are still needed for the future.

## Data Availability Statement

The original contributions presented in the study are included in the article/supplementary material, further inquiries can be directed to the corresponding author/s.

## Author Contributions

The author confirms being the sole contributor of this work and has approved it for publication.

## Conflict of Interest

The author declares that the research was conducted in the absence of any commercial or financial relationships that could be construed as a potential conflict of interest.

## Publisher's Note

All claims expressed in this article are solely those of the authors and do not necessarily represent those of their affiliated organizations, or those of the publisher, the editors and the reviewers. Any product that may be evaluated in this article, or claim that may be made by its manufacturer, is not guaranteed or endorsed by the publisher.
